# Effects of Hormone Agonists on Sf9 Cells, Proliferation and Cell Cycle Arrest

**DOI:** 10.1371/journal.pone.0025708

**Published:** 2011-10-03

**Authors:** Maeva Giraudo, Jérôme Califano, Frédérique Hilliou, Trang Tran, Nathalie Taquet, René Feyereisen, Gaëlle Le Goff

**Affiliations:** 1 Institut National de la Recherche Agronomique, UMR 1301 Interactions Biotiques et Santé Végétale, Centre National de la Recherche Scientifique, UMR 6243, Université de Nice Sophia Antipolis, Sophia-Antipolis, France; 2 UMR 6023 CNRS-Université Blaise Pascal, Bât. Biologie A – Campus des Cézeaux, Aubière, France; 3 Département des affaires réglementaires, Grasse, France; 4 Lanaud Gestion-Pôle de Lanaud, Boisseuil, France; 5 Bioimagerie, Villeneuve Loubet, France; New Mexico State University, United States of America

## Abstract

Methoxyfenozide and methoprene are two insecticides that mimic the action of the main hormones involved in the control of insect growth and development, 20-hydroxyecdysone and juvenile hormone. We investigated their effect on the *Spodoptera frugiperda* Sf9 cell line. Methoxyfenozide was more toxic than methoprene in cell viability tests and more potent in the inhibition of cellular proliferation. Cell growth arrest occurred in the G2/M phase after a methoprene treatment and more modestly in G1 after methoxyfenozide treatment. Microarray experiments and real-time quantitative PCR to follow the expression of nuclear receptors ultraspiracle and ecdysone receptor were performed to understand the molecular action of these hormone agonists. Twenty-six genes were differentially expressed after methoxyfenozide treatment and 55 genes after methoprene treatment with no gene in common between the two treatments. Our results suggest two different signalling pathways in Sf9 cells.

## Introduction

Growth and development are controlled by two major hormones in insects, the steroid 20-hydroxyecdysone (20E) and the sesquiterpenoid juvenile hormone (JH) [1]. The cross-talk between these two hormones regulates all stages from egg-larva-pupa to adult. A high level of 20E is required to initiate all developmental transitions and JH determines the nature of the moult [2]. JH is necessary for larval moulting and growth [3]. The signalling action of these hormones involves nuclear receptors. If the mode of action of 20E is well-known, that of JH remains more enigmatic. 20E exerts its action through binding to a nuclear receptor heterodimer consisting of an ecdysone receptor (EcR) and ultraspiracle (USP) which is the insect ortholog of retinoid-X-receptor from vertebrates [4]. The complex regulates expression of target genes by binding to gene promoter regions. In Drosophila, it was shown that 20E linked to its receptor activates early genes among which are the transcription factor regulators, the Broad complex (BR-C), E74 and E75 [5], [6]. It is those transcription factors that in turn regulate late genes that have direct effector roles (including affecting cell death, cellular proliferation, differentiation and cuticle production). Several receptor candidates for JH exist including MET (Methoprene tolerant) a member of the bHLH-PAS transcription factor family [7] and USP [8]. MET can bind JH at physiological concentrations [9] whereas USP was shown to bind JH with low affinity, at concentrations at least 100 times lower than expected for a nuclear receptor [10]. However the situation is complex and it is difficult to generalize findings on Met and USP from one insect group to another. Indeed, *Met* has a close paralog in Drosophila, germ cell expressed (*gce*) [11]; hence Met-null mutants are fully viable [12]. The *Met/gce* duplication is recent and the two paralogs are found in the Drosophila genus but are not found in mosquitoes [13]. In other insects, *Met* has only one ortholog and in *Tribolium castaneum* its depletion by RNAi causes premature pupal morphogenesis [14]. A phylogenetic study of USP receptors shows that there are two types of receptor in arthropods, one having lost the ability to bind a ligand as in *Bemisia tabaci* (Hemiptera) and *T. castaneum* (Coleoptera) and another still able to bind a ligand in Diptera and Lepidoptera [15]. Moreover, understanding of the molecular signalling mechanism downstream of JH binding to its putative receptor remains limited. Two transcription factors, the Broad complex (BR-C) and Krüppel homolog 1 (Kr-h1) seem to play an important role [16]–[19]. Minakuchi et al. (2009) have proposed a model in the red flour beetle *T. castaneum* whereby Kr-h1 works downstream of Met at the larval stage and downstream of Met but upstream of BR-C in the pupa, allowing the inhibition of metamorphosis in one case or its initiation in the other [20]. It was recently shown that the crosstalk between 20E and JH signalling pathways could be mediated by a nuclear receptor co-activator, the steroid receptor co-activator in *T. castaneum* and its homolog in the mosquito *Aedes aegypti* AaFISC [21], [22]. This receptor interacts with EcR and Met respectively in presence of each hormone; however, its own role in the regulation of hormone responses needs further studies.

These hormonal receptors (EcR, USP and Met) are also the target for insecticides which act by disrupting insect development. Agonist hormone insecticides are of growing interest because some have selective toxicity, they are potent against pest insects and less or non toxic for beneficial insects, mammals, fishes and birds [23]. Among 20E agonists are diacylhydrazines, a non-steroidal agonist family, having insecticide activity by binding to the EcR-USP receptors. This family of compounds provokes a premature moult that leads to the death of the insect and they are only acting on larvae. The activity spectrum of these compounds varies within insect orders and is directly correlated to receptor affinity for the insecticide [23], [24]. For example, methoxyfenozide is more effective against Lepidoptera [25]. The other insecticides that mimic hormone action are juvenile hormone agonists (JHA), initially designed to be metabolically stable JH analogs. Their precise molecular target is less well-known due to the still controversial mode of action of JH. JHA block insects at an intermediate stage during development, making them unable to emerge as normal adults. They also disrupt reproduction in insects where JH is gonadotropic. Methoprene was the first successfully used JHA [26] and it is more effective against dipteran insects compared to Lepidoptera.

Cell lines can be a useful tool to understand insecticide mode of action. Several members of the diacylhydrazines have been tested on insect cell lines, showing an inhibition of cellular proliferation. This is the case for the Drosophila Kc cell treated by RH-5849 and tebufenozide [27], [28]. Similar effects on cell growth arrest have also been observed with these compounds in the lepidopteran cell line IAL-PID2 from the imaginal wing disks of *Plodia interpunctella* [29], as well as in an epithelial cell line from *Chironomus tentans* [30]. Further studies on IAL-PID2 with tebufenozide have shown a G2/M arrest with an induction of mRNA transcripts for EcR and USP associated with a decrease in the expression of cyclin B, one of the proteins involved in cell cycle control [31]. Effects on cell proliferation were also reported for the JH agonists methoprene and fenoxycarb on IAL-PID2 [32], but the molecular mechanism leading to this arrest was not clarified.

In this study we were interested in the effects of insecticides that mimic hormone action on the *Spodoptera frugiperda* Sf9 cell line. The toxicity of methoxyfenozide and methoprene was evaluated. Both insecticides inhibit cellular proliferation. Flow cytometry analysis showed a distinctly different action between these compounds with a G2/M arrest after methoprene treatment, whereas methoxyfenozide induced a slight accumulation in G1. To investigate the differential molecular mode of action of these hormone agonists, we have performed microarray experiments and followed the expression of nuclear receptors by real-time quantitative PCR (RT-qPCR). Our results suggest two different signalling pathways in response to methoxyfenozide and methoprene treatments.

## Results

### Toxicological effects of methoxyfenozide and methoprene on Sf9 cells

**Figure 1:**
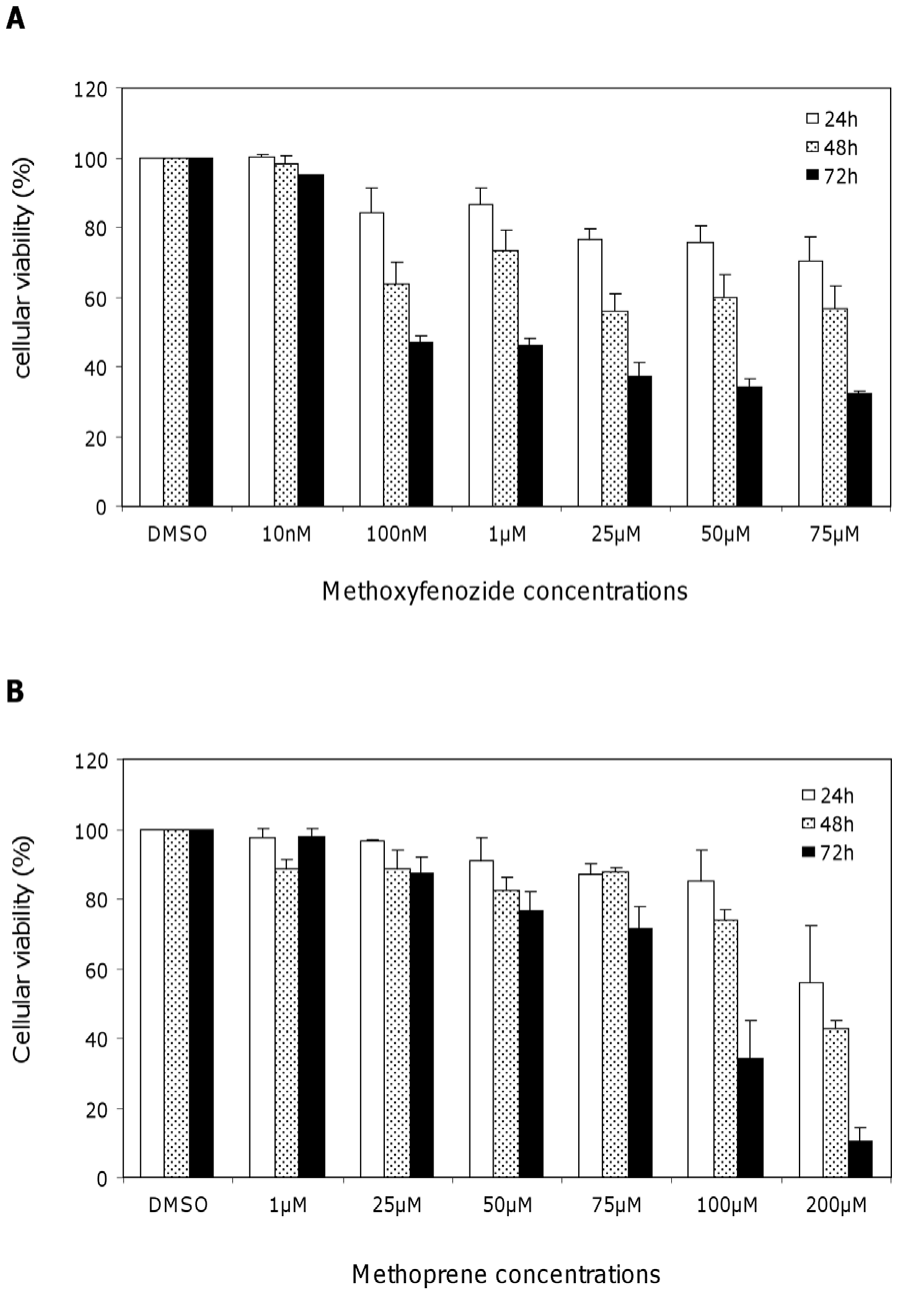
Methoxyfenozide and methoprene toxicity to Sf9 cells. Cell viability was followed by the MTT test at 24, 48 and 72 h post-treatment of Sf9 cells by methoxyfenozide (A) or methoprene (B). Data are the mean of three independent experiments with SE.

Cell viability was determined by the MTT test after an insecticide exposure of 24, 48 and 72 hours. Methoxyfenozide had almost no effect at 10 nM, the lowest concentration tested, but already a marked effect at 100 nM (Fig. 1A). Increased insecticide concentrations did not significantly modify the cell viability. The IC_50_ is below 100 nM at 72 h and cannot be calculated at 24 and 48 h. Figure 1B shows the results for methoprene. Almost no effect was observed up to 25 µM, whatever the length of treatment (90% of cells were viable). For a concentration range between 50 and 75 µM, toxicity was low (after 72 h, still 70% of cells were viable). The calculated IC_50_ at 48 and 72 h was 184.2±4.8 µM and 86.3±9.8 µM respectively. The two hormone agonists induced cell death and methoxyfenozide was more potent than methoprene by a toxicological factor of about 1,000.

### Cellular proliferation inhibition

**Figure 2:**
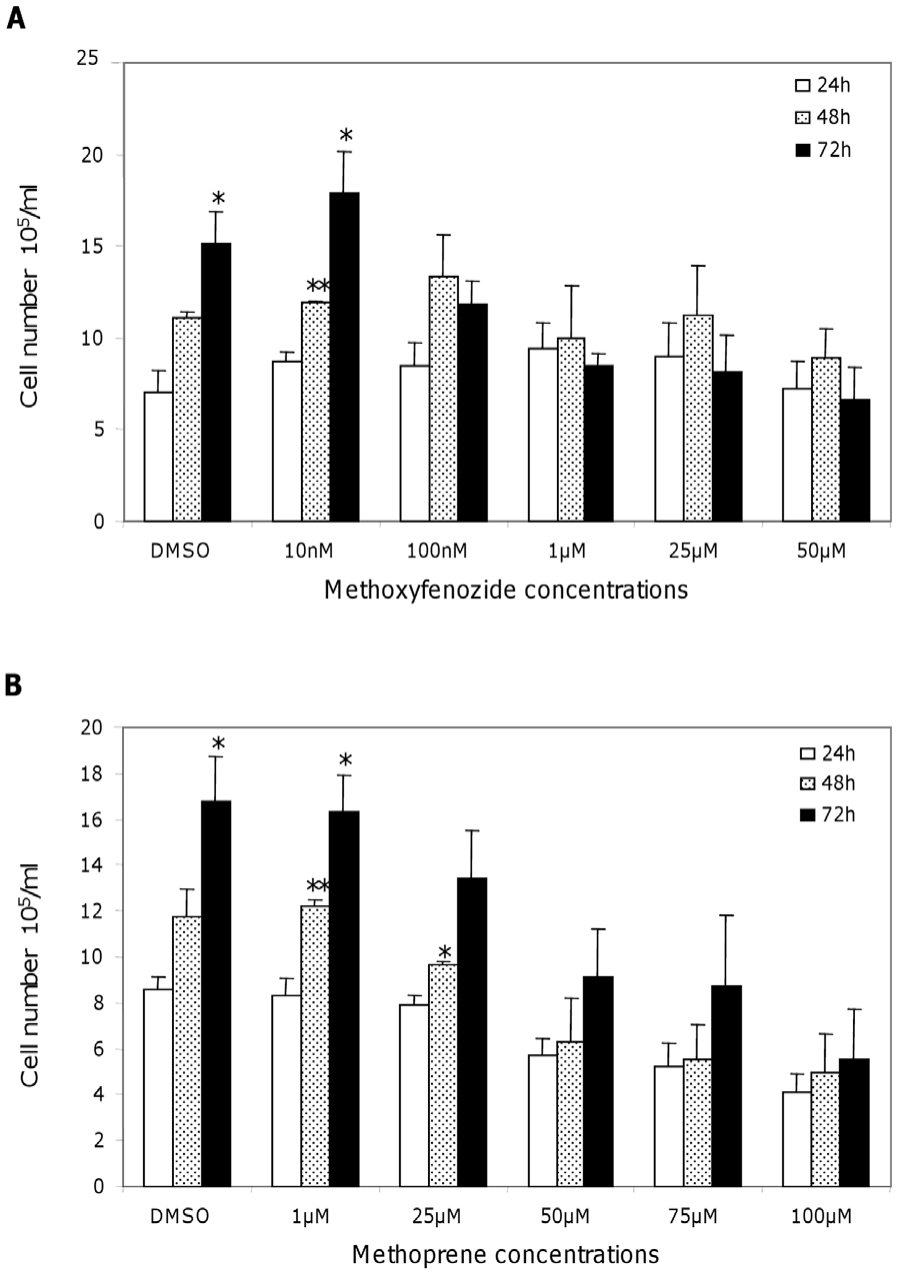
Effects of methoxyfenozide and methoprene on Sf9 cellular proliferation. Cells were counted 24, 48 and 72 h post-insecticide treatment, (A) methoxyfenozide, (B) methoprene. Data are the mean of three independent experiments with SE. A *t*-test was performed to determine significance of the results (* p-Value<0.05, ** p-Value<0.01).

The effect of these insecticides on cellular proliferation was monitored. Cells in the DMSO control grew to a density of 15 to 17.10^5^ cells/ml at 72 h (Fig. 2). Cell density at 24 h and 72 h in DMSO was significantly different, indicating that cells had proliferated. A normal and significant growth of the cells treated by methoxyfenozide was observed at the lower concentration (10 nM), however, all the other tested concentrations had an antiproliferative effect (Fig. 2A). At 50 µM of methoxyfenozide, the number of cells remained stable for 3 days. Methoprene had no effect at 1 µM with a cell density equivalent to the DMSO control. An inhibition of cell proliferation was observed in the concentration range between 50 and 100 µM, with no significant difference between numbers of cells at 24 h or 72 h (Fig. 2B). Proliferation arrest was reversible in both cases after removal of the insecticide (data not shown). Methoprene and methoxyfenozide therefore both caused an arrest of cell proliferation in a dose-dependent and reversible manner.

### Distinct phases of arrest in the cell cycle

In order to evaluate in which phase of the cell cycle the cells were arrested after insecticide treatment, we used flow cytometry. In cells treated with methoxyfenozide, a slight but significant (p-Value>0.01) accumulation occurred in the G0/G1 phase compared to the DMSO control (Table 1). In cells treated with methoprene, cells were arrested in the G2/M phase with 66.37% of cells in this stage (Table 1) and values in the three different phases were significantly different from the DMSO control. Cells were also treated with 25 µM methoprene acid as a negative control. Methoprene acid lacks the isopropyl ester group of methoprene and has therefore no JH agonist activity. Cell percentages in the cell cycle phases were similar in methoprene acid and DMSO-treated cells, indicating that methoprene acid did not affect the cell cycle at a concentration where methoprene does.

**Table 1:** Cell cycle analysis by flow cytometry after 24 h of insecticide treatment.

	G0/G1	S	G2/M	debris
0.4% DMSO control	21.77±0.86	14.85±0.44	51.48±2.14	11.78±1.02
Methoxyfenozide 100 nM	26.40±0.43*	13.23±0.33	47.13±0.78	13.17±1.03
Methoprene 25 µM	12.10±0.85*	9.4±0.95*	66.37±1.28*	12.13±0.39
Methoprene acid 25 µM	21.00±0.36	13.07±0.24	55.33±0.43	9.87±0.17

Data are expressed as mean ± SE (%) from triplicate measurements.

*p-Value<0.01; value significantly different to the DMSO control.

### Molecular pathways involved in the cell response to hormone agonist treatments

The phenotypic effects were similar between Sf9 cells treated by methoprene or methoxyfenozide with an arrest of the cell growth and cell morphology identical to that of control cells (data not shown). However, the insecticides had different molecular effects with an accumulation in the G0/G1 or G2/M phase for methoxyfenozide and methoprene, respectively. The molecular genetic regulation leading to this arrest still needs to be elucidated. We therefore compared the transcriptional effect of each hormone agonist by using a specifically designed oligonucleotide microarray. We chose the first concentration of insecticide having a significant effect on cell proliferation, i.e 100 nM for methoxyfenozide and 25 µM for methoprene, in order to focus on physiological effects and avoid toxicological effects.

Genes were considered as differentially regulated by the insecticide if their expression ratio was >1.5 or <0.66 and P value <0.05. Sequences of these genes were analysed in Blast2Go to assign them Gene Ontology (GO) terms and then classified in biological process level 3 (Table 2). The sequences for which no homology was found by blastx were submitted to a blastn in Butterflybase and classified in “hypothetical protein” category if homology was found with the sequence of another lepidopteran transcript, while the sequences restricted to *S. frugiperda* were put in a “hypothetical transcripts” category.

**Table 2:** Classification of genes regulated in Sf9 cells after treatment by methoxyfenozide or methoprene according to GO terms, blast2Go annotation level 3.

	methoxyfenozide		methoprene	
Biological process	up-regulated	down-regulated	up-regulated	down-regulated
biosynthetic process	2	1	4	3
cellular component organisation	3	0	1	3
cell cycle	0	0	1	1
cellular metabolic process	8	4	9	4
establishment of localization	2	1	3	1
multicellular organismal development	1	1	2	2
regulation of biological process	2	1	2	3
reproductive process	2	0	1	2
response to stress	0	2	5	2
transcription/translation	4	1	7	1
transport	2	1	2	1
hypothetical protein	0	2	4	0
hypothetical transcript	2	3	15	7
unknown function	0	1	0	0

After methoxyfenozide treatment, 26 genes were differentially expressed with 14 overexpressed and 12 down-regulated (Table 3). Looking in more detail at the list of differentially regulated genes may help to identify the potential function of genes involved in the molecular and cellular effects of methoxyfenozide. Therefore, genes were classified in more precise categories of Gene Ontology. The most up-regulated gene codes for a vacuolar ATPase subunit B that is involved in the transport of proton across the membrane. The most down-regulated gene is an aldehyde oxidase. Several genes with function in translation and transcription were overexpressed as well as genes encoding calcium-dependent proteins, like cadherin and calreticulin.

**Table 3:** Microarray data for selected genes after Sf9 treatment by methoxyfenozide.

Gene description and putative function	adhoc	ratio	P value
Actin cytoskeleton			
gelsolin	40151	1.59	0.0406
Carbohydrate metabolism			
6-phosphogluconolactonase	27223	2.39	0.0102
Catabolism process			
3-hydroxyisobutyryl Coenzyme A hydrolase	25654	0.64	0.0083
hydroxyphenylpyruvate dioxygenase	39567	1.64	0.0486
Cell adhesion			
cadherin	26462	6.32	0.0098
Chaperone proteins			
calreticulin	40650	4.47	0.0168
Proteolysis			
von Hippel-Lindau tumour suppressor protein	24976	0.18	0.0014
chymotrypsin	25557	2.9	0.0386
Reproductive protein			
vitellogenin	25793	1.69	0.0149
Response to stress/Detoxification			
heat shock protein 90	39008	0.57	0.0440
aldehyde oxidase	34664	0.14	0.0436
Sugar synthesis			
chondroitin sulfate synthase	44313	3.55	0.0254
Translation/Transcription			
polyadenylate binding protein 2	36158	6.19	0.0102
p27BBP/eIF6	33876	3.58	0.0252
dead box RNA helicase	25789	3.11	0.0422
Transport			
vacuolar ATPase subunit B	34648	9.44	0.0059
vacuolar ATPase subunit C	40849	0.64	0.0131

In the case of methoprene, 55 genes were differentially regulated with 39 over-expressed and 16 down-regulated (Table 4). There was no overlap between the genes regulated by methoxyfenozide and methoprene. The main category of genes up-regulated by methoprene is the hypothetical transcripts category (15 genes). The next categories are the genes involved in cellular metabolic process (9 genes) and transcription/translation (7 genes). These categories are also the most populated for cells treated by methoxyfenozide. Five genes upregulated by methoprene belonged to the response to stress category. The most induced (mitochondrial ribosomal protein L49) and most repressed (mitochondrial translational release factor 1 like) genes belong to the category of genes with functions in translation and transcription. This category has the highest number of genes regulated by methoprene (8 genes), followed by the class of genes with functions in response to stress and detoxification (7 genes). Several genes involved in spindle assembly are also over-expressed.

**Table 4:** Microarray data for selected genes after Sf9 treatment by methoprene.

Gene description and putative function	adhoc	ratio	P value
Amino acids biosynthetic process			
phosphoserine phosphatase	25880	0.62	0.0022
Extracellular matrix protein			
hemicetin like protein 1	25674	1.7	0.0355
Immune protein			
scolexin B like	34835	1.77	0.0116
Mitotic cell cycle checkpoint			
14-3-3 epsilon protein	41120	0.62	0.0022
Phospholipid biosynthetic process			
choline/ethanolamine kinase	36601	0.54	0.0273
Polyamine synthesis			
S-adenosylmethionine decarboxylase	44296	1.71	0.0151
Regulation of Rab GTPase activity			
Tbc1 domain family	39160	0.57	0.0026
Response to stress/Detoxification			
carboxylesterase	40600	3.89	3.75E-07
prophenoloxidase activating factor	26333	2.55	0.0086
aldehyde dehydrogenase 7 family member A1	25971	1.89	0.0019
pheromone degrading enzyme 2	34999	1.59	0.0172
apolipoprotein D precursor	35472	1.55	0.0119
DNAJ-1	38331	0.65	0.0456
uridine diphosphate glucosyltransferase	35495	0.55	0.0422
Spindle assembly			
microtubule associated protein RP/EB family 3	44445	2.86	0.0441
kinesin like protein	36395	2.75	0.0110
beta-tubulin cofactor E	36883	2.07	0.0152
Structural constituent of cuticule			
cuticle protein 1 like	38620	0.61	0.0462
Translation/Transcription			
mitochondrial ribosomal protein L49	27227	4.62	3.83E-06
60S ribosomal protein L31	25976	1.77	0.0019
coiled-coil-helix-coiled-coil helix domain containing 8	34522	1.67	7.20E-05
ribosomal protein L10	38355	1.55	0.0139
spt3 associated factor 42	41154	1.52	0.0044
mitochondrial translational release factor 1 like	40890	0.43	0.0035
tRNA splicing endonuclease 2	35120	1.9	1.29E-05
bip2 like	41062	1.5	0.0050
Transport			
phosphate transport protein	38062	1.85	0.0017
translocase of inner mitochondrial membrane	40155	0.61	0.0079
Vesicle trafficking			
exocyst complex component 6	36460	1.5	0.0340

### Expression of hormone receptors

**Figure 3:**
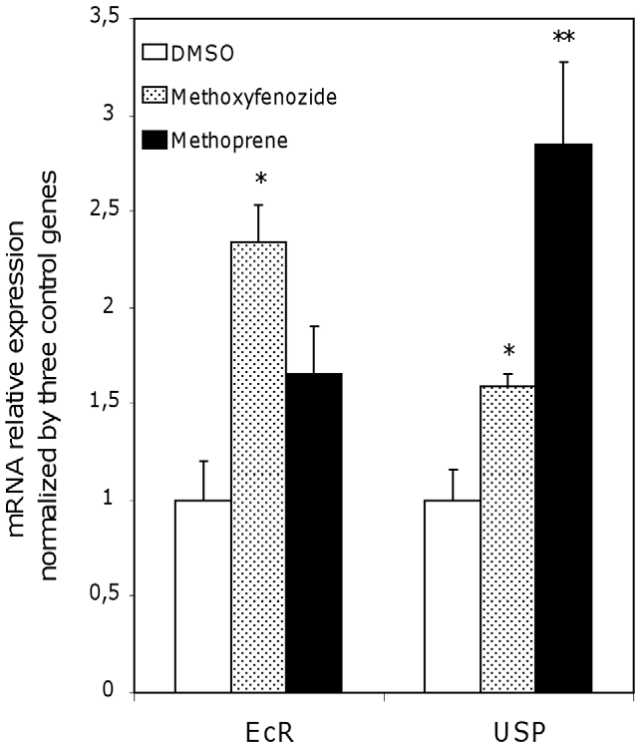
Transcript abundance of hormone nuclear receptors measured by RT-qPCR after insecticide treatment. A *t*-test was performed to determine significance of the results (* p-Value<0.05, ** p-Value<0.01).

Microarrays were not sensitive enough to detect transcripts of the nuclear receptors EcR or USP, and we did not have probes for Met on our array. Indeed we were unable to find the Met sequence in Spodobase (http://www.spodobase.univ-montp2.fr/Spodobase). Therefore, we used RT-qPCR approaches to study the expression of EcR and USP in Sf9 cells treated with methoxyfenozide or methoprene and compared to DMSO treated cells. Methoxyfenozide significantly induced the expression of both receptors, but methoprene only induced USP (Fig. 3).

## Discussion

Cell lines provide a useful tool to investigate the molecular mode of action of insecticides. The *Spodoptera frugiperda* Sf9 cell line is probably the most widely used for high level expression of recombinant proteins [33]. Moreover, *S. frugiperda* from which the Sf9 cell line is derived is a major crop pest, a polyphagous insect able to feed on many different host plants. We decided to use this cell line to determine the effects of two insecticides which are agonists of major hormones controlling insect development, 20E and JH.

Methoxyfenozide acts as an agonist of 20E by binding to the EcR/USP receptor complex. Its affinity for the receptor in Lepidoptera is 420 times higher than that of the insect moulting hormone [23], [25]. However, the affinity varies within insect orders, it is more potent on the lepidopteran cell line *Plodia interpunctella* than on the *Drosophila* Kc cells [23]. Methoprene has a different spectrum of activity, being very effective against dipteran insects but less so against Lepidoptera [34]. These different potencies are correlated to data obtained in our toxicological tests, where methoxyfenozide acts at a lower concentration than methoprene. However it seems that there are two populations of cells in the Sf9 line. One very sensitive to methoxyfenozide dies at doses 10 to 100 nM, which is in accordance to data obtained on another cell line Se4 from *Spodoptera exigua* [35], and another one more resistant for which stronger doses of 1 to 75 µM had no effect. Nevertheless these two putative cell sub-populations have the same sensitivity for methoprene.

We show that the two insecticides inhibit cellular proliferation. Treatment by tebufenozide, another diacylhydrazine, leads to the same effects in two other lepidoptera cell lines, IAL-PID2 from *Plodia interpunctella* [31] and Se4 from *Spodoptera exigua* [36]. Methoprene and another analog of JH, fenoxycarb, significantly inhibit cell proliferation of the IAL-PID2 cell line [32]. Auzoux-Bordenave *et al.* (2005) reported that tebufenozide arrested the cell cycle in G2/M in the same cell line. The moulting hormone itself, 20E, causes an arrest in G2 in Kc cell [37] and in IAL-PID2 cells [38] whereas arrest occurs in the G1 phase in mosquito C7-10 cells [39]. The accumulation of cells in a given phase of the cell cycle is usually reported 10 to 24 h after treatment by hormones or analogues [31], [39]. We chose to perform our flow cytometry and microarray experiments at 24 h. In Sf9 cells, we show that inhibition of cellular proliferation associated with methoxyfenozide treatment induces a modest accumulation in G1 phase. In contrast, methoprene blocks the cells in G2/M. Cell accumulation in G1 or G2 correspond to an arrest at the two cell cycle checkpoints: in one case cells are stopped before DNA synthesis (G1) and in the other, entrance in mitosis is prevented (G2/M). When tested at the same concentration as methoprene, methoprene acid has no effect on cellular proliferation, which suggests that methoprene arrest of the cell cycle is due to its role as hormone mimic.

We have investigated insecticide mode of action at the molecular level in order to understand pathways leading to this cellular arrest. We used a custom microarray consisting of 9,773 probes of *S. frugiperda*, which represents approximately 67% of the genome compared to the lepidopteran model genome of *Bombyx mori* [40]. Several studies have examined the effects of hormones on cell lines [41], [42]. In addition, Mosallanejad *et al.* [43] reported microarray data on *Drosophila* cell line S2 resistant to methoxyfenozide. However, to date no study has been performed by microarray on cell lines following hormone agonist treatment. In our study, very few genes are differentially expressed after treatment with methoxyfenozide (26 genes) or methoprene (55 genes). It may therefore correspond to a more physiological response linked to the regulation of cell cycle rather than a generalized stress response to the xenobiotic. The cell cycle consists of four distinct phases: in the G1 phase cells grow and cyclin D is expressed. In the S phase DNA replication occurs and cyclins E and A predominate. In the G2 phase, degradation of cyclin E and accumulation of cyclin B occur. This phase is followed by mitosis and cell division [44]. The progression through the cell cycle is controlled by cyclins and cyclin dependent kinases (CDK). The cyclins form complexes with CDK, CDK4 for cyclin D, CDK2 for cyclin E. Our array only has one probe for cyclin A, but none for other cyclins. No change in cyclin A levels was detected, although Mottier *et al.* reported a significant decrease in the expression of cyclin A and B after a 20E treatment of IAL-PID2 cells, the level of both cyclins remaining very low between 12 and 36 h post-treatment [38]. On the other hand, we observed some differentially expressed transcripts that are consistent with an arrest of the cell cycle: as overexpression of different genes involved in the spindle assembly after methoprene treatment. Indeed a biosynthetic step occurs during the G2 phase, mainly involving the production of microtubules which are required during the process of mitosis. Genes such as cadherin are overexpressed in our methoxyfenozide experiments and these are overexpressed in cells that had stopped to proliferate [45]. Among the genes affected by methoxyfenozide, we find genes coding for different subunits of vacuolar ATPase. They are differentially regulated, with subunit B upregulated and C downregulated. Insect vacuolar ATPase (V-ATPase) consists of two functional parts, the peripheral catalytic V1 complex composed of eight different subunits (from A to H) that hydrolyzes ATP and the integral membrane V0 complex consisting of four different subunits (a,c,d,e) that transports protons across the membrane [46]. These two parts can disassemble and reassemble depending on conditions which regulate V-ATPase activity [47], [48]. Both subunits can bind actin filament [49], [50], but subunit C is the only subunit that can be phosphorylated [51]. Another specificity of subunit C is its release in the cytosol upon dissociation of the two complexes [52], [53]. Clearly, subunit C has its own properties and can be a good candidate to mediate signalling pathway [54]. Opposite regulation of subunits B and C may therefore not be surprising. Moreover, promoter studies have revealed different regulatory elements between the two subunits' genes in *M. sexta* [48]. Down regulation of V-ATPase in apical globelet cell of *Manduca sexta* during moulting and starvation was also suggested [47], [55]. These data may indicate a possible involvement of hormone on the regulation of V-ATPase expression.

Genes and pathways involved in the various stages of the cell cycle progression were identified in a study on human HeLa cells using small interfering RNAs to target >95% of the protein coding genes [56]. Several concordant observations can be made between genes shown to be essential in that study and genes differentially regulated in our study. For example, several ribosomal proteins, kinesin, DNA-J have been shown to be essential for G2/M progression and are differentially regulated after methoprene treatment. Similarly, eIF, ATPase and dead box RNA helicase are essential in G1 phase and are differentially regulated after methoxyfenozide treatment.

More information concerning our microarray results (unknown genes) should be obtained with the forthcoming release of the *S. frugiperda* genome. Arrest of cell proliferation occurs at two checkpoints by distinct gene regulatory mechanism, at our level of sensitivity there are no genes in common. The recent discovery of a receptor co-activator able to bind EcR or Met depending on hormone concentration may explain why genes are up or down regulated: this co-activator may be a possible link for the cross-talk in these two signalling pathways [21], [22]. The presence of such a receptor co-activator remains unproven in the Sf9 cells. Further work is required to understand more precisely the mode of action of hormone agonists.

## Materials and Methods

### Cell culture

The Sf9 cells (from Invitrogen), derived from the pupal ovarian tissue of *S. frugiperda*, were cultured in a flask at 27°C in monolayer with the insect-Xpress protein free medium (Lonza). Cell density was determined by Malassez haemocytometer counts and cell viability was evaluated by methylene blue (1 mg/ml, v/v) staining. Prior to experiments, cells were seeded onto 6 well plates at 5.10^5^ cells/ml and left at 27°C for adhesion. Attached cells were then treated for 24 h with different concentrations of methoxyfenozide, methoprene, methoprene acid (all in 0.4% DMSO) or with 0.4% DMSO alone.

### MTT assay of cell viability

Sf9 cells were seeded in 96-well culture plates and treated for 24, 48 and 72 hours with increasing concentrations of methoprene and methoxyfenozide. Cells in culture were then loaded with MTT (0.5 mg/ml) and incubated at 27°C for 2 hours. Cell homogenates were used to measure absorbance at 570 nm using a microplate reader (SpectraMax, Molecular Devices).

### Cell cycle analysis

Cellular DNA content was determined by staining cells with propidium iodide and measuring fluorescence (FACSCalibur, Becton Dickinson). The Sf9 cells were incubated for 24 hours with methoxyfenozide or methoprene then resuspended and fixed on ice for 30 minutes with 70% ethanol/PBS (10 mM Na2HPO4, 138 mM NaCl, 2.7 mM KCl, pH 7.4). The fixed cells were incubated for 20 min at 37°C in a solution containing 50 µg/ml RNAse and 50 µg/ml propidium iodide. For each cell population, 10,000 cells were analysed by FACS and the percentage of cells in a specific phase of the cell cycle was determined with the propidium iodide DNA staining technique [57]. Cells were classified in G0/G1, S and G2/M phases depending on the intensity of the fluorescence peaks.

### RNA extraction

Total RNA was extracted from cells of a well of the 6 wells plate using Trizol Reagent (Invitrogen Life technologies). Extractions were performed on three independent biological replicates.

### Microarray experimental design

Our oligonucleotides were designed from 79148 ESTs sequences of eight different tissues of *S. frugiperda* (http://www.spodobase.univ-montp2.fr/Spodobase/). Using the assembly analysis (programme CAP3), we obtained 10,092 contigs and singletons from these ESTs. Our *S. frugiperda* microarray consists of 9,773 60-mers oligonucleotides synthesized by Sigma-Aldrich that were designed to match unique contigs or singletons and to suit our hybridization conditions (GC content average 46% and average Tm of 86.8°C). Each comparison consisted of six slides, three biological replicates hybridized with dye swap (fully balanced dye swap) and duplicate spots. cDNA were synthesized from 7 µg of total RNA and labelled with the dyes Cy3-dCTP and Cy5-dCTP (Amersham) using the ChipShot direct labeling system (Promega) according to the manufacturer's instructions. The microarray were hybridized with cDNA prepared as described by Le Goff *et al.* [58] and scanned using a GenePixPro scanner (Axon,version 3.01). Experimental data and associated microarray designs have been deposited in the NBCI Gene Expression Omnibus (GEO) (http://www.ncbi.nlm.nih.gov/geo/) under serie GSE30937 and platform record GPL8717 using Mediante database for data transfer [59].

### Data analysis

We used the Bioconductor suite of statistical packages [60]: *limma* [61] for our data analysis. The expression intensity was obtained by subtracting the background intensity from the foreground intensity for each non-flagged spot (all flagged spots were eliminated). The expression data were normalized by the use of the within-array normalization with the “loess method” and the between-array normalization using the “quantile method” [62]. The linear model for series of arrays and empirical Bayes method were then applied for assessing differential expression [63]. The false discovery rate of the p-value for multiple tests was controlled by using the Benjamini-Hochberg method. Differentially expressed genes were selected if the absolute value of log2-fold-change was greater than 1, the adjusted p-value below 0.01 and the average intensity greater than twice the average background. In order to provide an overall measure of evidence of differential expression, we used Fisher's method for combining adjusted p-values from independent tests of significance of duplicate spots [64].

### Quantitative real-time PCR

Total RNA (1 µg) was reverse transcribed using the iScript cDNA Synthesis kit (Biorad). RTqPCR reactions were carried out on an Opticon monitor 2 (Biorad) using the qPCR Mastermix plus for SYBR Green I no ROX (Eurogentec). The PCR conditions were as follows: 50°C for 2 min, 95°C for 10 min, followed by 40 cycles of 95°C for 30 s, 60°C for 30 s and 72°C for 30 s. Each reaction was performed in technical triplicates and the mean of the three independent biological replicates was calculated. All results were normalized using mRNA level of three control genes (RpL4, L18 and G6PD) and relative expression values were calculated in R using the RqPCRAnalysis package developed in our laboratory (Hilliou and Tran, manuscript in preparation). Primer sequences and PCR efficiencies are listed in the Table 5.

**Table 5:** Primers used in RT-qPCR.

Name	primer sequence	fragment length	PCR efficiency %
EcR-F	5′-AAGCCTTTACCGGAGGATGT-3′	82	95
EcR-R	5′-TACCAGCGTCGTCTCATGTC-3′		
USP-F	5′-CGTATCAACCGACCTCCACT-3′	135	102
USP-R	5′-CCAGCTCAAGGGAACTGAAG-3′		
G6PD-F	5′-GGCCCTGTGGCTAACAGAAT-3′	142	99
G6PD-R	5′-CATCGTCTCTACCAAAAGGCTTC-3′		
L18-F	5′-CGTATCAACCGACCTCCACT-3′	126	104
L18-R	5′-AGGCACCTTGTAGAGCCTCA-3′		
RpL4-F	5′-CAACAAGAGGGGTTCACGAT-3′	149	99
RpL4-R	5′-GCACGATCAGTTCGGGTATC-3′		
